# Oxidative Stress in Renal Health

**DOI:** 10.3390/antiox14020144

**Published:** 2025-01-26

**Authors:** Ana Cristina Simões e Silva

**Affiliations:** Interdisciplinary Laboratory of Medical Investigation, Unit of Pediatric Nephrology, Department of Pediatrics, Faculty of Medicine, Universidade Federal de Minas Gerais (UFMG), Belo Horizonte 30130-100, Brazil; acssilva@hotmail.com; Tel.: +55-31-999870077

## 1. Introduction

Oxidative stress is caused by the increase in reactive oxygen species (ROS) and reactive nitrogen species (RNS) inside cells [1,2]. ROS include the superoxide anion, hydrogen peroxide, and hydroxyl radicals. Among these molecules, the superoxide anion is the main one responsible for oxidative stress in the kidney, being predominantly synthesized by the enzyme NOX-4, an isoform of nicotinamide adenine dinucleotide phosphate-oxidase (NADPH-oxidase) [3]. Enzymatic and non-enzymatic systems are responsible for the removal of ROS. The enzymatic system includes superoxide dismutase (SOD), catalase, glutathione peroxidase, glutathione reductase, glutathione S-transferase, peroxiredoxin, and thioredoxin, whereas the non-enzymatic system comprises ascorbic acid, alpha-tocopherol, carotenoids, flavonoids, and reduced glutathione [4]. Since ROS can damage lipids, proteins, and DNA, the antioxidant systems are critical for cell survival and function.

Many pathophysiological mechanisms related to chronic kidney disease (CKD), including uremic toxins, inflammation, and metabolic and hormone alterations, increase oxidative stress [4,5]. The excessive production of ROS and RNS associated with the reduction in antioxidant mechanisms can lead to the progression of CKD. Accordingly, the kidneys of animals with CKD induced by 5/6 nephrectomy had a high expression of NADPH-oxidase, low concentration of SOD, and increased levels of systemic nitrotyrosine [6]. In experimental models of polycystic kidney disease and diabetic and hypertensive nephropathies, the enzyme NOX-4 is upregulated in kidney tissue with consequent superoxide synthesis and mitochondrial injury [7,8,9]. The role of nuclear factor erythroid 2-related factor 2 (Nrf2), a transcription factor that stimulates the production of antioxidant molecules, was also investigated in experimental studies. The deficiency of Nrf2 increased oxidative stress and deteriorated diabetic kidney disease in *Akita* mice [10]. On the other hand, the administration of antioxidant molecules has been investigated in experimental studies and in patients with renal diseases. As an example, the compound bardoxolone, a synthetic Nrf2 activator, resulted in a persistent increase in glomerular filtration rate in patients with CKD stage 4 and type 2 diabetes [11].

This Special Issue reviews the complex mechanisms of oxidative stress in kidney disease and presents new evidence on the role of oxidative stress in experimental models, promising therapies that inhibit the production or action of ROS in kidney tissue, and a randomized controlled trial using curcumin in CKD patients.

## 2. Overview of Published Articles

The review of Frak and co-workers [Contribution 1] summarized the main mechanisms by which uremic toxins, oxidative stress, and renal fibrosis lead to the progression of CKD and also discussed the effects of well-known and new medications in renal diseases. The first topic of the article was about the clinical consequences of uremic toxin accumulation in CKD, which can lead to renal function deterioration, the development of atherosclerosis, or the increased incidence of cardiovascular events. The second topic reported the interactions between oxidative stress and inflammation, the mechanisms of ROS generation in acute kidney injury, the role of mitochondrial dysfunction in CKD, and the clinical consequences related to these biological processes. The third topic explained the mechanisms and pathways responsible for renal fibrosis. The next part of the article discussed the effects on renal diseases of well-known treatments including Glucagon-like Peptide-1 (GLP-1) agonists, Sodium–Glucose Cotransporter Protein 2 (SGLT2) inhibitors, and Sacubitril/Valsartan, as well as new drugs such as Finerenone and Canakinumab. In the last topic, the authors concluded that understanding the pathophysiological mechanisms of CKD is essential for the development of targeted therapeutic interventions.

In the context of new therapies, Lee and co-workers [Contribution 2] provided a comprehensive review about the pathways of oxidative stress and inflammation in CKD, the beneficial renal effects of treatments for metabolic dysfunctions, and the potential role of natural antioxidants in kidney diseases. The effects of ROS in CKD include oxidative post-translational modifications that activate latent transforming growth factor beta (TGF-β), which, in turn, promotes fibrosis via canonical Smad signaling and non-canonical JNK, and nuclear factor kappa B (NF-κB) pathways. ROS may also stimulate the intrinsic and extrinsic apoptosis pathways in renal cells. The second part of the article summarized studies about the beneficial effects of Statin, Metformin, GLP-1 agonists, SGLT2 inhibitors, Angiotensin-Converting Enzyme Inhibitors, and Angiotensin Receptor Blockers. The next topic was about the anti-inflammatory and antioxidant effects of bioactive supplements and herbs, including lactoferrin, *Boerhaavia diffusa*, *Amauroderma rugosum*, and *Ganoderma lucidum*, in experimental models of renal diseases. The authors concluded that clinical trials are necessary to confirm the promising findings obtained with natural antioxidant compounds in experimental studies.

Wang and Zhang [Contribution 3] reviewed the role of oxidative stress specifically in diabetic kidney disease (DKD). The importance of DKD as a common complication of diabetes mellitus and the main etiology of CKD was emphasized. Hyperglycemia stimulates the synthesis and release of ROS, the main sources of which are mitochondrial production, NADPH oxidases, uncoupled endothelial nitric oxide synthase, xanthine oxidase, cytochrome P450, and lipoxygenase. Hyperglycemia also activates mechanisms that impair antioxidant activity. Consequently, high levels of ROS trigger downstream signaling pathways related to inflammation and fibrosis such as phosphoinositide 3-kinase/protein kinase B (PI3K/Akt), TGF-β/p38-mitogen-activated protein kinase, nuclear factor kappa B (NF-κB), adenosine monophosphate-activated protein kinase, and Janus kinase/signal transducer and activator of transcription (JAK/STAT) signaling. The antioxidant properties of medications already used in clinical practice and new promising treatments for DKD were also discussed. The authors concluded that multitarget therapeutic strategies to reduce oxidative stress, inflammation, and fibrosis are still necessary for DKD.

The role of oxidative stress in cystic kidney diseases was also reviewed [Contribution 4]. Subbash and co-workers [Contribution 4] first explained the subtypes of cystic kidney diseases with emphasis on polycystic kidney disease (PKD) and its main type, the autosomal dominant PKD. The next part of the review discussed the effects of ROS in the pathogenesis of PKD. The upregulation of NOX-4 in PKD produces ROS, which inhibit nitric oxide (NO) synthase with a consequent depletion of NO; oxidize mitochondrial DNA, proteins, and lipids; impair cell function; and reduce ATP production. These mechanisms lead to hypoxia, overactive mitophagy, apoptosis, fibrosis, and inflammation in kidney tissue. Biomarkers of oxidative stress are increased early in patients with autosomal dominant PKD before the occurrence of arterial hypertension. The authors reported the effects of therapeutic interventions used in clinical practice such as xanthine oxidase inhibitors and tolvalptan, as well as experimental treatments including mitochondrial antioxidant MitoTEMPO, ketone b-hydroxybutyrate, and the ketogenic diet. They concluded that NOX4 and xanthine oxidase inhibitors, mitochondrial antioxidants, dietary modifications, and tolvaptan are promising treatments for PKD. However, randomized controlled trials are still necessary to evaluate the efficacy and safety of these interventions in patients with cystic kidney diseases.

Liu and co-workers [Contribution 5] performed an overview on the role of Nrf2 and its activating compounds in systemic lupus erythematosus (SLE) and lupus nephritis (LN). The excessive production of oxidants and reduced levels of antioxidant stimulate inflammation and produce immune system dysfunction, worsening LN. Under oxidative stress conditions, Nrf2 stimulates the transcription of enzymes and antioxidant and anti-inflammatory molecules and indirectly inhibits NF-κB by reducing the activation of the NLRP3 inflammasome. In turn, high levels of NF-κB reduce the coactivator CBPB/p300 necessary for the production of Nrf2. Experimental studies show the renoprotective effects of Nrf2 activation in LN. The authors also discussed the potential therapeutic role of natural products and synthetic compounds that activate Nrf2 in SLE. The Nrf2 activators include dimethyl fumarate, sulforaphane, artemisinin, polyphenols, triterpenoids, and bardoxolone. On the other hand, the abnormal activation of Nrf2 or high doses of activators may result in side-effects. The conclusion was that further studies are necessary to investigate the complex mechanisms beyond Nrf2 effects and to explore potential therapeutic targets for SLE and LN.

Still concerning autoimmune conditions affecting kidney tissue, Bruschi and co-workers [Contribution 6] provided a perspective article about the potential role of antibodies against antioxidant enzymes in autoimmune glomerulonephritis and in antibody-mediated graft rejection. The authors initially made a historical overview of ROS in renal diseases and then explained the mechanisms of membranous nephropathy and LN, showing the importance of oxidative stress in both conditions. The next topic reported the clinical relevance of anti-SOD antibodies. Antibodies against the antioxidant enzyme SOD2 have been detected in the blood and kidney tissue of patients with LN and membranous nephropathy. The presence of anti-SOD2 antibodies may represent an abnormal response to oxidative stress and result in poor clinical outcomes. Post-transplant glomerulopathy is another example of an autoimmune condition in which antibodies against the antioxidant enzymes glutathione S-transferases (GSTs) have been associated with antibody-mediated reaction and graft rejection. The potential antioxidant approaches including diet adjustments, use of nutrients (olive oil), natural substance supplementation (curcumin, Chinese herbs, and celastrol), and antioxidant medications (acetyl cysteine, melatonin, and others) were also commented on. The authors suggested the use of molecules with antioxidant action in autoimmune conditions and the monitoring of circulating levels of antibodies against SOD and GST.

Three experimental studies investigated potential therapies in animal models of acute kidney injury (AKI) [Contribution 7, Contribution 8, Contribution 9]. Airik and co-workers [Contribution 7] evaluated the role of JP4-039 in mice with cisplatin-induced acute kidney injury. The study had four experimental groups: group 1 (control, vehicle injection), group 2 (cisplatin injection), group 3 (cisplatin injection and 24 h JP4-039 administration by gavage), and group 4 (gavage administration of JP4-039 and, 1 h later, cisplatin injection). The administration of JP4-039 before or after cisplatin injection improved renal function, and reduced kidney tissue damage, tubular injury, interstitial fibrosis, inflammatory markers expression, ROS production, and the apoptosis and ferroptosis of tubular cells. The conclusion was that JP4-039 is a promising therapy and deserves further investigation in additional experimental models [Contribution 7]. Yubolphan and co-workers [Contribution 8] examined the effects of berberine administration in aged rats with sepsis-associated AKI. Male Wistar rats aged 26 months were allocated into four groups: group 1 (control—sham surgery), group 2 (sepsis induced by cecal ligation puncture—CLP), group 3 (CLP and oral gavage of 25 mg/kg of berberine for 5 days), and group 4 (CLP and oral gavage of 50 mg/kg of berberine for 5 days). Both doses of berberine reduced serum levels of malonyldialdehyde (MDA), TNF-α, creatinine, blood nitrogen urea, and Neutrophil Gelatinase-Associated Lipocalin (NGAL). Berberine administration (25 and 50 mg/kg) also attenuated kidney tissue injury, mitochondrial damage, and ROS production in kidney tissue and reduced the activation of toll-like receptor 4/NF-κB and NLRP3 inflammasome pathways. The authors concluded that clinical trials with berberine are needed to confirm its efficacy and safety in humans [Contribution 8]. Schiffer and co-workers [Contribution 9] evaluated the effect of a novel NOX-4 inhibitor, GLX7013114, in a mouse model of ischemia–reperfusion (IR)-induced AKI. Male C57BL/6J mice aged 5 months were allocated to four groups: sham-operated group, AKI 3d group (animals submitted to IR and evaluated 3 days after reperfusion), AKI 3d + NOX4i 24 h (24 h administration of NOX4 inhibitor during reperfusion in animals submitted to IR and evaluated 3 days after reperfusion), and AKI 3d + NOX4i 76 h (76 h administration of NOX4 inhibitor during the 3-day reperfusion period in animals submitted to IR). The administration of NOX4 inhibitor for 76 h reduced apoptosis and improved glomerular filtration rate, tubular injury, and mitochondrial function. The effect of the 24 h administration of NOX-4 inhibitor was not able to increase glomerular filtration rate and reduce tubular damage, but prevented apoptosis and improved mitochondrial function. The authors suggested that the chronic inhibition of NOX-4 might have a role in preventing AKI in clinical situations associated with IR [Contribution 9].

In another experimental study, Guo and co-workers [Contribution 10] investigated the in vivo and in vitro effects of ADP-ribosylation factor-interacting protein 2 (ARFIP2) in the mitophagy and autophagy of podocytes in diabetic nephropathy. For in vitro analyses, the authors employed an *ARFIP2*-deficient immortalized podocyte cell line using the CRISPR/Ca technique, while a mouse model of Streptozotocin-induced type I diabetes was used for in vivo experiments. *ARFIP2* deficiency in immortalized human podocytes hindered autophagy and compromised mechanisms related to mitochondrial function, resulting in the impaired fission of mitochondria and increased mitophagy. In diabetic animals, the deficiency of ARFIP2 also impeded autophagy and produced a foot process effacement of podocytes, kidney tissue injury, and albuminuria. The authors hypothesized that *ARFIP2* deficiency compromises the regulation of autophagy and mitophagy with the consequent deterioration of kidney function in diabetic nephropathy.

Gimblet and co-workers [Contribution 11] performed a randomized controlled trial to test the effects of curcumin supplementation on the vascular and cognitive function of patients with CKD. A total of 88 adults with CKD stages 3b or 4 took part in a randomized, double-blinded, placebo-controlled trial for one year. Vascular function was assessed by brachial artery flow-mediated dilation, nitroglycerin-mediated dilation, and carotid–femoral pulse wave velocity, while the NIH Toolbox Cognition Battery was used to evaluate cognition. The comparison between curcumin supplementation and placebo for 12 months did not show significant differences in both vascular and cognitive function. The authors concluded that the results do not corroborate the indication of curcumin supplementation for vascular and cognitive alterations. However, further studies are necessary to investigate the role of curcumin in inflammation and CKD progression.

## 3. Conclusions

Homeostasis between oxidative molecules and antioxidant mechanisms is essential for renal health. Experimental and clinical studies support the pathophysiological role of oxidative stress in several kidney diseases. ROS interact with inflammatory and fibrogenic molecules, leading to the deterioration of renal function and kidney tissue injury. Some medications used in clinical practice produce beneficial effects by stimulating antioxidant pathways or inhibiting the formation of ROS. Several natural and synthetic compounds have also been tested in experimental models of renal diseases. However, randomized controlled trials are scarce in this research field.

This Special Issue included five review articles showing important mechanisms of oxidative stress and therapeutic possibilities for CKD, DKD, PKD, and LN. The role of antibodies against antioxidant enzymes in autoimmune glomerulonephritis and in antibody-mediated graft rejection was discussed in a perspective article. In addition, three experimental studies tested the compounds JP4-035, berberine, and GLX7013114 in animal models of AKI and showed renoprotective effects. Another experimental study found that ARFIP2 modulates the autophagy and mitophagy of podocytes in diabetes nephropathy, and its deficiency resulted in the deterioration of kidney function. Finally, a randomized controlled trial did not find differences between curcumin supplementation and placebo in improving the vascular and cognitive function of CKD patients. In conclusion, oxidative stress is a critical mechanism for kidney tissue damage and an important therapeutic target for renal diseases. Further studies are still necessary to translate experimental findings to clinical practice.
